# Oxidative stress gradient in a medium during human corneal organ culture

**Published:** 2012-06-16

**Authors:** Siv Johnsen-Soriano, Kristiane Haug, Emma Arnal, Cristina Peris-Martinez, Morten C. Moe, Francisco Javier Romero, Bjørn Nicolaissen

**Affiliations:** 1 Fundación Oftalmológica del Mediterráneo (FOM), Valencia, Spain; 2 School of Medicine, Universidad Católica de Valencia, Spain; 3 Centre for Eye Research, Department of Ophthalmology, Oslo University Hospital and University of Oslo, Norway

## Abstract

**Purpose:**

Lipid peroxidation content was measured in an organ culture medium after one-week storage of human donor corneas. Moreover, the effects of the medium on oxidative stress, antioxidant capacity, and the proliferation of cultured human corneal cells were studied.

**Methods:**

The medium was sampled from the upper and lower halves of storage vials and from controls (n=42). Malondialdehyde (MDA) was measured by high pressure liquid chromatography (HPLC). Cultured human corneal epithelium (CRL-11515) was exposed to different medium samples and monitored for changes in MDA (enzyme-linked immunosorbent assay [ELISA]), total antioxidant capacity (antioxidant assay kit), and proliferation (Ki-67).

**Results:**

A significant increase in MDA was observed in the organ culture medium in the lower level of storage vials. The addition of this fraction to cultured cells increased MDA significantly after 3 days, and the medium from both levels significantly increased MDA after 7 days. The medium from both levels significantly decreased the total antioxidant capacity of the cells but did not affect proliferative activity.

**Conclusions:**

An oxidative gradient with an evident biologic effect is established in the medium in vials during organ culture of human donor corneas. Donor tissue stored at the bottom or in lower levels of such vials is exposed to a significant amount of oxidative stress.

## Introduction

Corneal transplantation using donor corneas obtained after storage in an eye bank is the most common of all transplant procedures. In the US, donor corneas are maintained in a medium at 4 °C, while most European eye banks use the organ culture system in which donor corneas are maintained in a medium at 31 °C/32 °C. Clinical results are similar when comparing this method with storage in Optisol-GS (Chiron Intraoptics, Irvine, CA) at 4 °C and reflect the high quality of these systems [1]. They have been used in clinics worldwide for more than 30 years. However, although corneal transplant has an acceptable success rate (30%–90% depending on the disease that causes the need for a transplant), donated corneas are often simply not available in most developing countries. Every year in Europe, 40,000 blind people are put on a corneal transplant waiting list. Therefore, new strategies for improving human donor corneal storage to optimize the available material are crucial.

Both storage systems represent a stressful environment for the donor tissue. During organ culture, cell death and loss depend on the condition of the tissue [2,3] and on factors related to the storage procedure, such as incubation time, type of medium, amount of serum, and temperature [4-7]. Relatively little information is available regarding the various types of insults and molecular damage initiating the chains of events resulting in apoptosis or in other types of cell death during organ culture storage. However, in cell cultures of human corneal endothelium, sensitivity to oxidative stress has been linked to the type of medium during incubation at 37 °C [8], and during “cold” storage, there is a progressive increase in levels of nitric oxide breakdown products in the medium [9].

In the present study, we sampled organ culture medium after one-week storage of human donor corneas and examined the accumulation of malondialdehyde (MDA), a lipid peroxidation breakdown product and a commonly used marker for oxidative stress. The effects of the medium on antioxidant defense mechanisms, the oxidative damage of lipids, and the proliferation of cultured human corneal epithelial cells were also examined. Due to the known accumulation of debris at the bottom of such storage vials and variations in procedures regarding the positioning of donor corneas in such vials [10], medium from the upper levels and medium from the lower levels of the vials were analyzed separately. The biologic effect of such an “aging” organ culture medium has not, to our knowledge, been evaluated. Such information could add relevant insight to discussions on routines regarding positioning of donor corneas and medium changing during organ culture storage.

## Methods

### Medium

**Figure 1:**
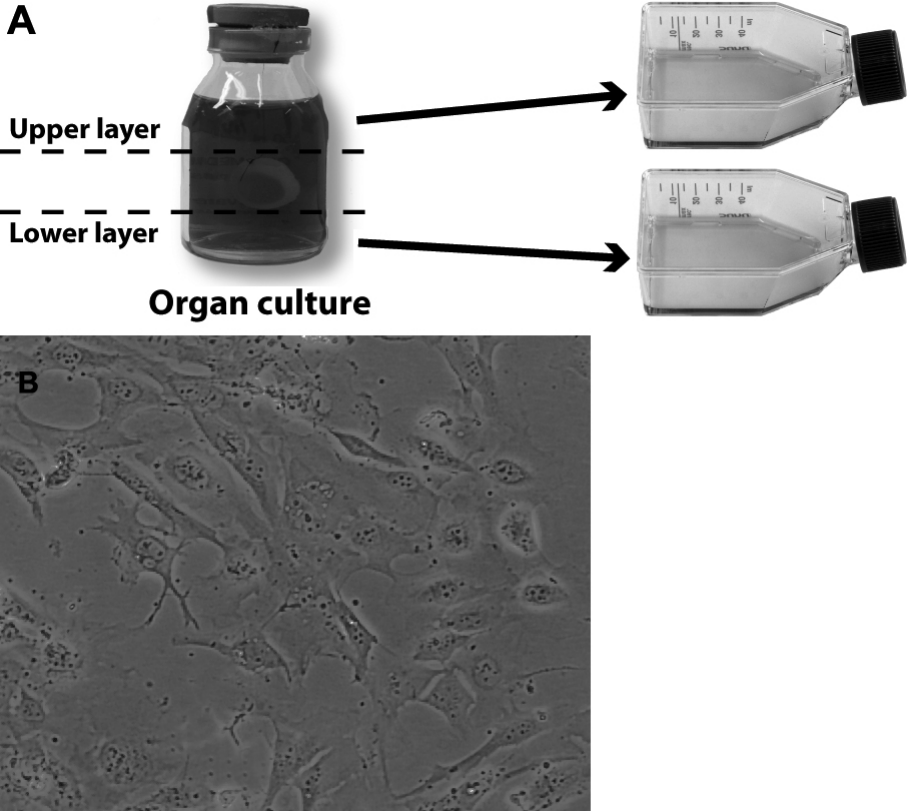
Experimental setup. **A**: Organ culture medium was collected from the upper and lower levels of the storage vials after 7 days and analyzed for MDA. **B**: Subconfluent human corneal epithelial cell cultures were exposed to medium from the different levels and to control medium for 0, 3, and 7 days before analysis.

The Norwegian Eye Bank, Oslo University Hospital, Oslo, Norway, stores corneas at 32 °C in organ culture before surgery. The organ culture medium was prepared by the hospital pharmacy and consisted of Minimal Essential Medium (MEM) with Earle’s salts and L-glutamate (Gibco, Invitrogen, Paisley, UK), sodium hydrogen carbonate (2.20 μl/ml), HEPES buffer (2.98 μg/ml), 8% heat-inactivated fetal calf serum, amphotericin B (5 μg/ml), gentamicin (50 μg/ml; Sigma Aldrich, St. Louis, MO), and Vancomycin (100 μg/m; Alpharma ApS, Kobenhavn, DK), pH 7.1–7.2. Corneas aimed for transplantation were sutured and placed in the middle of a 50-ml closed sterile storage container with 50 ml organ culture medium. Samples of medium (15 ml) from 42 containers were obtained from the lower and upper halves of vials in which donor corneas had been stored for 7 days and from fresh control medium (Figure 1). All samples were stored at −85 °C before analytical procedures or assays on cultured cells.

### HPLC

MDA was measured in the medium by high-pressure liquid chromatography (HPLC) according to a modification of the method of Richard et al. as previously described [11]. Briefly, MDA was measured by HPLC (Waters-LC Module I Plus; Waters Cromatografia SA, Spain) on a Spheryc-5 column (ODS 5 μM, 250×4.6 mm; Brownlee-Columns; Waters Cromatografia SA) at the flow rate of 1 ml/min.

### Cell line and treatment

The human corneal epithelial cell line CRL-11515 (#CRL-11515; American Type Culture Collection, LGC/ATCC, Middelsex, UK) was maintained in D-MEM:F12 (1:1) GlutaMAX (Invitrogen Life Technologies Corporation, Grand Island, NY) supplemented with 10% heat-inactivated fetal bovine serum, 2.5 μg/ml Amphotericin B, 100 units/ml Penicillin, and 100 μg/ml streptomycin (all Sigma-Aldrich) in a humidified incubator at 32 °C with 5% CO_2_. The cells were grown in six-well plates (Nunc; Thermo Fisher Scientific, Boston, MA), and after 3 days, the growth medium was exchanged with either fresh organ culture medium (control) or with organ culture medium obtained from the upper or lower levels of the storage containers. The medium was changed every second day. Cells were harvested for analytical procedures on day 0 and after 3 and 7 days.

### Immunohistochemistry

For each time point (day 0, 3, and 7), cells were scraped off and centrifuged (300× g, 10 min). The supernatant was removed and the pellet resuspended in 2 drops of plasma (Oslo blood bank, Ullevål, Norway) and 1 drop of thrombin (Sigma-Aldrich) to make a gel. The gels were prepared for immunohistochemical staining by being fixated in 4% fresh formaldehyde and embedded in paraffin. Three-micrometer sections were cut and stained for the proliferation marker Ki-67 (SP6, 1:200; Thermo Fisher Scientific) using Benchmark Classic (Ventana Medical Systems, Inc., Tucson, AZ) and a standard peroxidase technique with diaminobenzidine as chromogen (ultraView Universal DAB detection kit; Ventana, Tucson, AZ). The proliferation index was evaluated by three independent observers.

### Enzyme-linked immunosorbent assay (ELISA)

For lipid peroxidation measurement in cultured cells, the cells were scraped off and centrifuged (300× g, 10 min) before the supernatant was removed and the cell pellets were stored at −85 °C. The assay was performed using a commercial kit (Lipid Peroxidation Microplate Assay Kit; Oxford Biomedical Research, Rochester Hills, MI) following the manufacturer’s instructions. This assay is based on the reaction of two molecules of the chromogenic reagent N-methyl-2-phenylindole with one molecule of malondialdehyde (MDA) at 45 °C to yield a stable chromophore. Levels of MDA were monitored by reading the absorbance at 586 nm, where the absorbance is proportional to the concentration.

The antioxidant capacity was measured with a commercial kit (Antioxidant Assay Kit; Cayman, Ann Arbor, MI) following the manufacturer’s instructions. Briefly, the assay is based on the ability of the antioxidants in the sample to inhibit the oxidation of 2,2'-Azino-bis(3-ethylbenzothiazoline-6-sulfonic acid) (ABTS) to ABTS+ by metmyoglobin. The capacity of the antioxidants in the sample to prevent ABTS oxidation is compared with that of Trolox, a water-soluble tocopherol analog, and is quantified as molar Trolox equivalents. The amount of ABTS+ can then be monitored by reading the absorbance at 405 nm, which is proportional to its concentration.

### Statistical analysis

Data are expressed as means±SEM. Comparisons between groups were done using one-way ANOVAs and were followed by Bonferroni multiple comparison tests. Statistical differences were set at the p<0.05 level.

## Results

### Medium

**Figure 2:**
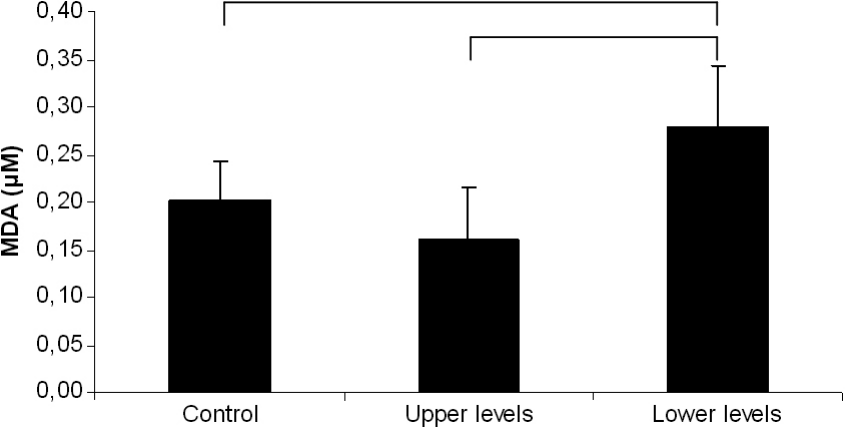
Lipid peroxidation products were significantly elevated in organ culture medium collected from the lower level of the vials where the donor corneas had been stored for 7 days when compared to medium from the upper level and from fresh control medium. Statistical analysis by one-way ANOVA showed significant differences between the groups (p=0.000) and was followed by the Bonferroni multiple comparison test (p<0.05 between the groups linked by square brackets).

The content of the lipid peroxidation marker MDA was measured in the upper and lower levels of 7-day-old organ culture medium after storage of donor corneas aimed for transplant purposes. The analysis showed that the level of MDA was significantly increased in the lower half of the container when compared to levels detected in the upper half and in control samples (Figure 2).

### Cell culture assay

**Figure 3:**
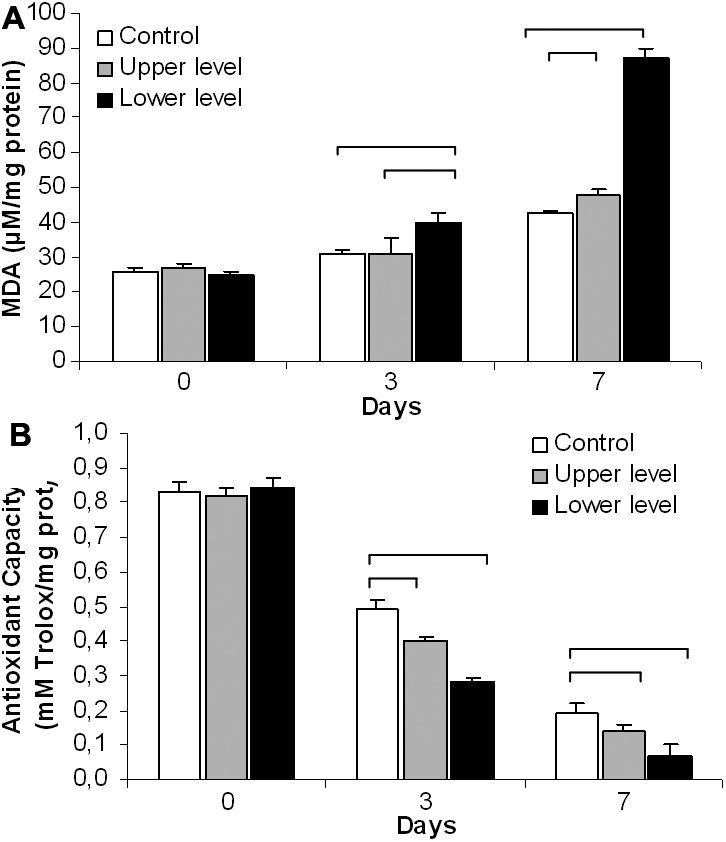
Oxidative stress markers. **A**: Lipid peroxidation products were significantly elevated in the epithelial cells after 3 days of exposure to lower level of organ culture medium. After 7 days of exposure, both groups (upper and lower levels) were significantly different from the control. Statistical analysis by one-way ANOVA showed significant differences between the groups (p=0.000) and was followed by the Bonferroni multiple comparison test (p<0.05 between the groups linked by square brackets). **B**: The total antioxidant capacity was found to be significantly reduced in both upper and lower levels when compared to the control after 3 days of exposure. This tendency continued, and after 7 days of exposure, both upper and lower levels were further reduced with respect to the control. Statistical analysis performed by one-way ANOVA showed significant differences between the groups (p=0.000) and was followed by the Bonferroni multiple comparison test (p<0.05 between the groups linked by square brackets).

Lipid peroxidation products were significantly elevated in the cultured epithelial cells after 3 days of exposure to organ culture medium obtained from the lower level of the storage vials when compared to the control and the upper level groups. After 7 days, the cultures maintained in the medium from the lower as well as from the upper levels were significantly increased compared to the control group (Figure 3A).

Total antioxidant capacity was measured and was found to be significantly reduced in cells maintained in the medium from both upper and lower levels when compared to the control after 3 days of exposure, and a further significant reduction was detected after 7 days (Figure 3B).

The proliferating activity was estimated by K_i_-67 staining of paraffin sections of the different groups, but no significant difference was found. However, the percentage of K_i_-67 staining cells was slightly higher in the control medium (80%) when compared with cultures grown in the upper (70%) and lower (60%) levels of the medium (data not shown).

## Discussion

The main purpose of the donor corneal storage systems is to provide an environment that ensures cellular viability and low levels of cellular and molecular damage. In the present study, we wanted to examine levels of lipid peroxidation breakdown products in the medium used for donor corneal organ culture. We found that an oxidative gradient with a significant effect on cellular antioxidant defense and lipid constituents appears in the vials as early as during the first week of incubation.

Oxidative stress is a relative overload of oxidants caused by increased free radical production and/or decreased antioxidant defense systems [12]. MDA is a lipid peroxidation breakdown product resulting from such an overload, and the production of this aldehyde is used as a biomarker for the level of oxidative stress [13]. By crosslinking with amino groups, MDA forms covalent protein adducts referred to as advanced lipoxidation end products. Such oxidative reactions may interfere with basic cellular functions such as the synthesis of proteins and with proliferation. These reactions are further linked to the progression of tissue damage in various ocular metabolic, degenerative, and inflammatory processes [13-17], and oxidative stress has been implicated in the progression of corneal disorders including keratoconus, bullous keratopathy, and Fuchs’ endothelial dystrophy [18].

Less is known about the effect of oxidative stress on human corneal cells during organ culture storage. Oxidative stress in cultured murine corneal cells results in the depletion of anti-oxidative vitamins, rising levels of MDA, and ultimately cell death [15]. The sensitivity to oxidative stress depends on the incubation medium, and it may be increased in the commonly used MEM medium [8]. Furthermore, oxidative stress mediated by H_2_O_2_ reduces proliferation in corneal endothelial cells in a dose-dependent manner [14]. Our findings demonstrate that one-week “exhausted” medium from organ cultures causes an early and significant decay in cellular antioxidant defenses. The early effect on cellular MDA was significant only for cells incubated in the medium from the lower level of the storage vials, and this confirms the presence of an oxidative gradient in the organ culture medium. It might seem like a contradiction that the lipid peroxidation does not increase in the upper level in the medium, whereas the cells exposed to the upper level samples have increased MDA concentrations and decreased antioxidant capacities. However, the same is true for the control cells, and this might be due to a natural and gradual decline of the cells conserved in the corneal storage medium, which has a limited storing time. The effect on proliferation was not significant. We examined epithelial cells known to proliferate more overtly in organ culture [19,20] than the endothelium [14,21], and the effect on endothelial cells remains to be elucidated. However, a significant repair of endothelial defects is known to occur during the first week of organ culture of human corneas [22].

The protocols for positioning of the donor cornea in the storage vial and for intervals between medium changes vary between eye banks [7,10], and our observations support previous recommendations on eye bank routines regarding organ culture of human donor cornea.

As for the positioning of the donor cornea in the vials, there is a known accumulation of debris at the bottom of storage vials, and this is most probably a key factor in the generation of the observed oxidative gradient. Due to this accumulation, positioning of the donor tissue in a hanging suture or in a floating device has been recommended [10]. Data herein show that donor corneas at the bottom or in lower levels of vials are exposed to medium with significantly higher levels of oxidative potential than corneas that are positioned in the upper half by aid of a hanging suture or a floating device.

Regarding medium changes, there is a decrease in glucose concentration in the medium and in the tissue and decay in the oxygen consumption during organ culture. Furthermore, there is an increase in lactate concentration, and the pH shows a gradual decline from 7.36 to 6.64 during a four-week incubation [23,24]. Continuous incubation of corneal epithelial cell cultures without medium changes has been shown to induce cell death [25]. Our findings show that the oxidative potential in the medium is relevant in both upper and lower levels of the vials after one week of incubation. Although the exact role and influence of oxidative stress during the storage of human donor corneal tissue remains uncertain, the findings herein support the recommendation of medium changes every week [23].

## References

[1] Rijneveld WJ, Remeijer L (2008). van RG, Beekhuis H, Pels E. Prospective clinical evaluation of McCarey-Kaufman and organ culture cornea preservation media: 14-year follow-up.. Cornea.

[2] Borderie VM, Sabolic V, Touzeau O, Scheer S, Carvajal-Gonzalez S, Laroche L (2001). Screening human donor corneas during organ culture for the presence of guttae.. Br J Ophthalmol.

[3] Builles N, Kodjikian L, Burillon C, Damour O (2006). Major endothelial loss from corneas in organ culture: importance of second endothelial count.. Cornea.

[4] Crewe JM, Armitage WJ (2001). Integrity of epithelium and endothelium in organ-cultured human corneas.. Invest Ophthalmol Vis Sci.

[5] Møller-Pedersen T, Hartmann U, Moller HJ, Ehlers N, Engelmann K (2001). Evaluation of potential organ culture media for eye banking using human donor corneas.. Br J Ophthalmol.

[6] Sperling S (1979). Human corneal endothelium in organ culture. The influence of temperature and medium of incubation.. Acta Ophthalmol (Copenh).

[7] Ayoubi MG, Armitage WJ, Easty DL (1996). Corneal organ culture: effects of serum and a stabilised form of L-glutamine.. Br J Ophthalmol.

[8] Jäckel T, Knels L, Valtink M, Funk RH, Engelmann K (2011). Serum-free corneal organ culture medium (SFM) but not conventional minimal essential organ culture medium (MEM) protects human corneal endothelial cells from apoptotic and necrotic cell death.. Br J Ophthalmol.

[9] Meisler DM, Koeck T, Connor JT, Aulak KS, Jeng BH, Hollyfield JG, Stuehr DJ, Shadrach KG (2004). Inhibition of nitric oxide synthesis in corneas in storage media.. Exp Eye Res.

[10] Lie JT, Lock FM, Mulder PG (2008). van der WJ, Melles GR. Floating device for donor corneas in organ culture.. Br J Ophthalmol.

[11] Romero MJ, Bosch-Morell F, Romero B, Rodrigo JM, Serra MA, Romero FJ (1998). Serum malondialdehyde: possible use for the clinical management of chronic hepatitis C patients.. Free Radic Biol Med.

[12] Finkel T, Holbrook NJ (2000). Oxidants, oxidative stress and the biology of ageing.. Nature.

[13] Del Rio D, Stewart AJ, Pellegrini N (2005). A review of recent studies on malondialdehyde as toxic molecule and biological marker of oxidative stress.. Nutr Metab Cardiovasc Dis.

[14] Joyce NC, Zhu CC, Harris DL (2009). Relationship among oxidative stress, DNA damage, and proliferative capacity in human corneal endothelium.. Invest Ophthalmol Vis Sci.

[15] Serbecic N, Beutelspacher SC (2005). Anti-oxidative vitamins prevent lipid-peroxidation and apoptosis in corneal endothelial cells.. Cell Tissue Res.

[16] Johnsen-Soriano S, Garcia-Pous M, Arnal E, Sancho-Tello M, Garcia-Delpech S, Miranda M, Bosch-Morell F, Diaz-Llopis M, Navea A, Romero FJ (2008). Early lipoic acid intake protects retina of diabetic mice.. Free Radic Res.

[17] Ahuja-Jensen P, Johnsen-Soriano S, Ahuja S, Bosch-Morell F, Sancho-Tello M, Romero FJ, Abrahamson M, van Veen T (2007). Low glutathione peroxidase in rd1 mouse retina increases oxidative stress and proteases.. Neuroreport.

[18] Arnal E, Peris-Martínez C, Menezo JL, Johnsen-Soriano S, Romero FJ (2011). Oxidative stress in keratoconus?. Invest Ophthalmol Vis Sci.

[19] Joseph A, Powell-Richards AO, Shanmuganathan VA, Dua HS (2004). Epithelial cell characteristics of cultured human limbal explants.. Br J Ophthalmol.

[20] Slettedal JK, Lyberg T, Ramstad H, Beraki K, Nicolaissen B (2007). Regeneration of the epithelium in organ-cultured donor corneas with extended post-mortem time.. Acta Ophthalmol Scand.

[21] Slettedal JK, Lyberg T, Roger M, Beraki K, Ramstad H, Nicolaissen B (2008). Regeneration with proliferation of the endothelium of cultured human donor corneas with extended postmortem time.. Cornea.

[22] Nejepinska J, Juklova K, Jirsova K (2010). Organ culture, but not hypothermic storage, facilitates the repair of the corneal endothelium following mechanical damage.. Acta Ophthalmol.

[23] Redbrake C, Salla S, Frantz A, Reim M (1999). Metabolic changes of the human donor cornea during organ-culture.. Acta Ophthalmol Scand.

[24] Hjortdal JO, Ehlers N, Andersen CU (1989). Some metabolic changes during human corneal organ culture.. Acta Ophthalmol (Copenh).

[25] Kim JM, Stapleton F, Willcox MD (1999). Induction of apoptosis in human corneal epithelial cells in vitro.. Aust N Z J Ophthalmol.

